# Late syphilis presenting with extensive granulomatous cutaneous and pulmonary involvement

**DOI:** 10.1016/j.idcr.2026.e02638

**Published:** 2026-06-17

**Authors:** Ilona Shurmelova, Angela Oellinger, Helmut J.F. Salzer, Drolaiz H.W. Liu, Emmanuella Guenova, Wolfram Hoetzenecker

**Affiliations:** a Department of Dermatology and Venereology, Kepler University Hospital, Linz, Austria; b Division of Infectious Diseases and Tropical Medicine, Department of Internal Medicine 4, Kepler University Hospital, Linz, Austria; c Medical Faculty, Johannes Kepler University, Linz, Austria; d Ignaz-Semmelweis-Institute, Interuniversity Institute for Infection Research, Vienna, Austria; e Department of Pathology and Molecular Pathology, Kepler University Hospital, Linz, Austria; f Department of Pathology, GROW Research Institute for Oncology and Reproduction, Maastricht University Medical Center, Maastricht, the Netherlands; g Clinical Department of Immunodermatology, Kepler University Hospital, Linz, Austria; h Clinical Research Institute for Inflammation Medicine, Kepler University Hospital, Linz, Austria

**Keywords:** Syphilis, Late syphilis, Gummatous syphilis, Pulmonary syphilis, Cutaneous granuloma

## Abstract

**Introduction:**

Syphilis is a protean infection capable of mimicking various dermatological and systemic diseases, often complicating diagnosis in its later stages.

**Case presentation:**

A 26-year-old HIV-negative man with a history of inadequately monitored syphilis presented with headache, cough, and widespread erythematous plaques with central atrophy. Investigations revealed an RPR titer of 1:128 and positive CSF treponemal serology. Chest CT demonstrated peripheral nodular lung consolidations, and ophthalmologic exam showed signs of prior uveitis. A skin biopsy confirmed late-stage syphilis, revealing extensive dermal tuberculoid granulomas and gummatous necrosis. Following a 14-day course of intravenous penicillin G, the pulmonary lesions completely resolved, cutaneous lesions markedly improved, and the RPR titer appropriately declined at the six-month follow-up.

**Conclusion:**

This case illustrates a rare presentation of late syphilis with concurrent pulmonary involvement and widespread granulomatous skin lesions, emphasizing the need for clinicians to maintain a high index of suspicion for atypical manifestations of Treponema pallidum.

## Case Ilustrated

A 26-year-old man presented with widespread erythematous plaques featuring central atrophy affecting the entire integument, including the scalp, but sparing the genital and oral mucosa (Fig. A, B). Concurrently, he exhibited patchy alopecia, a diffuse headache, and a nonproductive cough. Four years prior, the patient had been diagnosed with syphilis and treated with three doses of intramuscular benzathine penicillin G. At that time, his rapid plasma reagin (RPR) titer was 1:8; however, no subsequent serological follow-up was documented. The patient reported ongoing high-risk sexual behavior.

**Figure:**
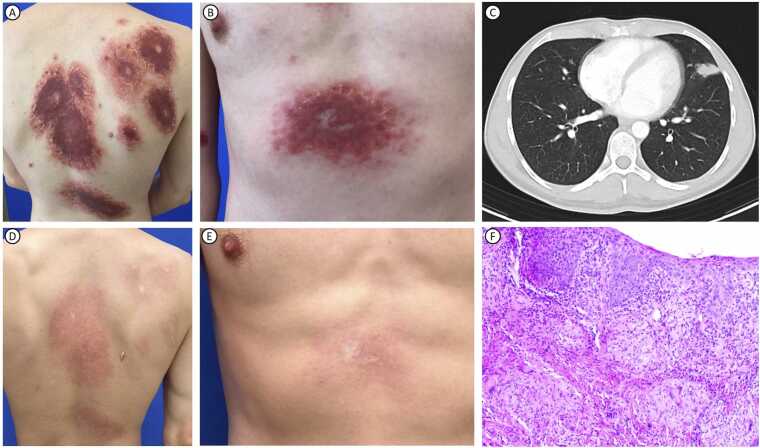
Photographs of skin lesions on the patient’s back (A) and chest (B) before and after the treatment (D), (E). (C) Peripheral nodular consolidations in left lower lung lobe in the computer tomography scan. (F) Hematoxylin and eosin-stained section of a skin biopsy showing superficial and deep granulomatous dermatitis and multinucleated Langhans-type giant cells.

Current serological evaluation revealed an RPR titer of 1:128 and a positive *Treponema pallidum* hemagglutination assay (TPHA), consistent with an active infection. Due to the neurological symptoms, a lumbar puncture was performed.

Cerebrospinal fluid (CSF) analysis revealed a positive TPHA and a non-reactive venereal disease research laboratory (VDRL) test; therefore, central nervous system involvement could not be definitively ruled out. Chest computed tomography (CT) demonstrated peripheral nodular consolidations in both lower lung lobes (Figure C). Human immunodeficiency virus (HIV) screening was negative. Cardiac magnetic resonance imaging (MRI) showed no evidence of myocarditis or myocardial scarring. An ophthalmologic consultation revealed posterior synechiae in the right eye, consistent with prior anterior uveitis of potential syphilitic etiology. A biopsy of the cutaneous lesions demonstrated a superficial and deep dermal inflammatory infiltrate with extensive tuberculoid granulomas, numerous multinucleated giant cells predominantly of the Langhans type, and focal gummatous necrosis (Figure F). As is typical for late-stage syphilitic lesions, immunohistochemistry did not reveal T. pallidum. The patient received intravenous aqueous crystalline penicillin G at a dose of 30 million units daily for two weeks. At the six-month follow-up, the cutaneous lesions had markedly improved (Figure D, E) and the pulmonary lesions had completely resolved. The RPR titer declined to 1:32 at six weeks and to 1:8 at six months, confirming an adequate serological response.

Our case highlights the importance of recognizing atypical cutaneous and systemic manifestations of syphilis. It illustrates an unusual presentation of late-stage syphilis characterized by widespread granulomatous cutaneous lesions and pulmonary involvement, underscoring the classic 'protean' nature [1] of this infection and the necessity of maintaining a high index of clinical suspicion.

## CRediT authorship contribution statement

**Wolfram Hoetzenecker:** Conceptualization, Supervision, Validation, Writing – review & editing. **Ilona Shurmelova:** Conceptualization, Investigation, Validation, Visualization, Writing – original draft, Writing – review & editing. **Angela Öllinger:** Validation, Writing – review & editing, Investigation. **Helmut J.F. Salzer:** Investigation, Validation, Writing – review & editing. **Drolaiz H.W. Liu:** Investigation, Validation, Writing – review & editing. **Emmanuella Guenova:** Investigation, Validation, Writing – review & editing.

## Consent

Written informed consent for publication was obtained from the patient.

## Ethical approval

Not applicable.

## Funding sourses

This work did not receive any specific grant from funding agencies in the public, commercial, or not-for-profit sectors.

## Declaration of Competing Interest

The authors declare the following financial interests/personal relationships which may be considered as potential competing interests: HJFS reports personal honoraria from Gilead, Pfizer, MSD, Bistrol Myres, Insmed, and GSK outside the submitted work; DL received honorarium from AstraZeneca not related with the submitted work; IS, WH, EG and AO have no conflicts of interest. If there are other authors, they declare that they have no known competing financial interests or personal relationships that could have appeared to influence the work reported in this paper.
